# Study on the Particle Deposition Characteristics of Transpiration Cooling Structures with Sintered Wire Mesh

**DOI:** 10.3390/mi15040452

**Published:** 2024-03-28

**Authors:** Zhe Zhang, Xiang Luo, Yubo Peng

**Affiliations:** 1 School of Energy and Power Engineering, Beihang University, Beijing 100191, China; dream070141@buaa.edu.cn; 2 Research Institute of Aero-Engine, Beihang University, Beijing 100191, China; xiang.luo@buaa.edu.cn

**Keywords:** transpiration cooling, sintered woven wire mesh structure, particle deposition, time evolution law, spatial distribution law

## Abstract

Transpiration cooling based on a porous structure has an ultra-high cooling efficiency, which is expected to be one solution to improve the cooling technology of aero-engine turbine blades. However, particulate impurities in the gas flow channel continue to deposit on the surface of turbine components, blocking cooling holes, which causes great harm to the cooling of turbine blades. In this study, a sintered metal mesh plate was selected as the transpiration cooling structure, and the evolution of particle deposition quality and deposition thickness on the transpiration cooling surface with time, as well as spatial distributions of particle deposition thickness at different times, were explored through experimental and simulation methods. The results showed that, with the increase in spray time, deposition quality and maximum deposition thickness of the transpiration cooling surface gradually increased. Along the main-stream direction, when spray time was short, deposition thickness was higher in a narrow range upstream of the experimental specimen. With the increase in spray time, deposition thickness gradually decreased along the direction of the transpiration cooling mainstream. In the spanwise direction, when spray time was very short, deposition thickness in the spanwise direction was more consistent and, after spray time increased further, the deposition thickness distribution began to tend to a “∩”-type distribution. It can be seen from the simulation results of the metal wire mesh particle deposition that particles were easily deposited on the windward side of the metal wire in the main-stream direction, which agreed with the experimental distribution characteristics of the metal wire mesh deposition. Moreover, the increase in blowing ratio reduced the deposition of particles on the wall of the metal wire mesh.

## 1. Introduction

With the continuous pursuit of improving the overall performance characteristics of aero-engines, such as thrust–weight ratio and efficiency, the temperature before the turbine has greatly increased [1], and a new requirement has been put forward for the cooling technology of turbine blades: to use as little cooling air as possible to meet the growing cooling demand. However, traditional cooling technologies, such as impingement cooling and film cooling, are inadequate. For example, impingement cooling causes significant pressure loss, which will greatly increase the temperature gradient of the cooled area and then generate thermal stress. There are also many technical problems in film cooling, such as the reasonable arrangement of hole spacing. When the distance between holes is small, it leads to significant stress concentration and, eventually, causes material fracture. When the distance between holes is large, the requirements for cooling become higher [2,3]. The process of film cooling is greatly affected by the external flow state. If the heat load in the region is high, such as in the leading edge of the blade, the actual cooling effect is not good [4]. If the flow of the region is good, the cooling efficiency is affected by compressibility and unsteadiness [5,6]. Therefore, in order to meet the demand for the efficient cooling of high-performance aero-engines in the future, it is necessary to make new technological changes in turbine blade cooling technology.

**Figure 1:**
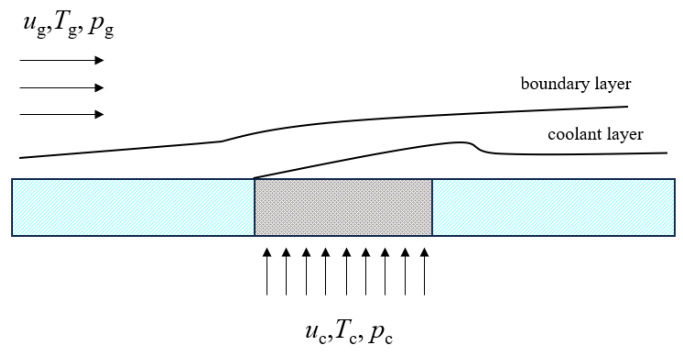
Schematic diagram of divergent cooling technology.

Divergent cooling is an efficient cooling method. Research on its technology began in the 1940s [7] and was first applied to rocket engines. In 1990, National Aeronautics and Space Administration (NASA) summarized several new cooling schemes and pointed out that divergent cooling was the most promising cooling method. Since then, divergent cooling technology has received more attention in various aerospace powers and is considered to be the most likely cooling technology to address the extremely harsh thermal environment of the liquid rocket engine thrust chamber and to provide thermal protection for the key components of hypersonic vehicles. The divergent cooling of porous media can be regarded as a limiting method of film cooling, with a small pore size and a dense pore distribution (see Figure 1, arrows on the left indicate mainstream gas, arrows below indicate cold gas). The cooling medium flows out from micropores of the porous material in the opposite direction of the heat flow, and the cooling medium is uniformly transported to the whole high-temperature surface using the good heat transfer performance of the porous material. The cooling medium exchanges heat with the porous material, which absorbs the heat and reduces the overall temperature of the porous medium skeleton. At the same time, a continuous and evenly distributed film structure is formed on the high-temperature surface, which separates the high-temperature surface from the main-stream gas, thereby reducing the surface temperature of the hot end and effectively improving the cooling efficiency and coverage area of the cooling medium. This cooling method has the theoretically optimal cooling efficiency of all cooling methods.

Although divergent cooling has a high cooling efficiency, its application for aero-engine turbine blades faces some practical problems. The most important problem is the deposition of particles on the walls of the turbine blade [8]. The particle deposition has a variety of effects on the turbine blade, mainly including: (1) External particle impurities are inhaled from the engine inlet, some of which invade the cooling channel of the turbine blade through the engine air system and easily deposit in and block the narrow internal cooling channel. This results in a decrease in the cooling efficiency of the turbine blade, which in turn increases the temperature of the turbine blade. This will affect the service life, working performance, and safe operation of an aero-engine [9]. When the cooling medium contains particle impurities, the particle flow impinges on the inner wall of the blade to form particle deposition. The fluid temperature, impact velocity, particle concentration, and particle size all affect the particle deposition on the inner wall of the blade [10]. In addition, the deposition of particles in the cooling medium will also block film holes [11,12]. (2) The impact of particles on the blade leads to the erosion of hot end components, resulting in the permanent loss and irreversible damage of blade wall material. The result of this process is a permanent deterioration of turbine performance and increased maintenance and repair costs [13]. Therefore, predicting the particle deposition and erosion rate in the high-pressure turbine flow channel and the deposition morphology of the blade wall surface to estimate the aerodynamic loss of the blade after deposition is a popular topic in the field of turbine blade particle deposition research [14]. (3) Sand particles in the gas are deposited on the hot Thermal Barrier Coating (TBC) surface. On the high-temperature TBC surface, deposits are converted into molten calcium–magnesium alumino-silicate (CMAS) compounds [15]. CMAS compounds penetrate the coating through pores, and the yttria-partially stabilized zirconia (YSZ) coating dissolves and re-precipitates into zirconia particles, causing the coating to peel off. In addition, TBC impregnated with CMAS compounds exhibits a low strain tolerance, making it prone to thermomechanical failure during repeated heating and cooling cycles (thermal cycles). Therefore, when the divergent cooling technology is introduced into the cooling of turbine blades, it is necessary to consider pollutant particles in the molten state under the action of high temperatures and the particle deposition distribution when encountering the divergent cooling area with lower temperatures. An in-depth understanding of the mechanism of interactions between particle deposition and divergent cooling plays an important role in the design of engine turbine blades.

Particulate matter deposition is the main mode for the deposition, wear, and corrosion of engine high-pressure turbine blades. Understanding the interactions between particle deposition and the turbine blade cooling structure is particularly important for reducing the influence of particle deposition and even inhibiting it. Many researchers have focused on this. Friedlander and Johnstone [16] performed particle deposition analysis for the motion of small particles, with a particle size range of 0.8 μm ≤ *d*_p_ ≤ 2.6 μm in a vertical circular tube. The results showed that, when the Stokes numbers of particles were less than 1, the deposition of particles was related to turbulent diffusion. When there was a particle concentration gradient along the normal direction of the wall near the wall, the movement of particles in the normal direction of the wall was analogous to the turbulent diffusion of the fluid along the normal direction of the wall near the wall. The velocity gradient of the fluid near the wall was large, and velocity fluctuations along the normal direction of the wall led to the exchange of momentum and mass between fluid layers at different heights in the normal direction of the wall. The turbulent fluctuation velocity in the normal direction of the wall was also the main reason for the exchange of momentum and mass between different particle concentration layers at different heights in the normal direction of the wall, which in turn led to the deposition of particles on the wall.

Raj [17] introduced a series of particle deposition experiments on divergent cooling turbine blades using fuel contaminated with alumina or fly ash (maximum particle size less than 6.3 μm) under the typical operating conditions of gas turbines (average turbine inlet temperature of 1427 °C, main-stream flow rate of 0.95 kg/s), and discussed the effect of electrostatics on particle deposition. H. Kozlu and J. F. Louis [18,19] from the Massachusetts Institute of Technology studied the effect of divergent cooling on particle motion and analyzed factors such as the divergent cooling blowing ratio, particle diameter, and particle density. In the experiment, glass particles with a particle size range of 5–30 μm were used to simulate the trajectories of dust particles with a particle size range of 0.5–3 μm in the actual engine, by matching the Stokes number of actual engine particles. It was observed that, when the blowing ratio F was greater than 2.46%, there was a region where the particle concentration near the wall was 0. With the increase in particle diameter and particle density, the particle concentration near the wall increased and the thickness of the particle concentration boundary layer decreased. Jensen et al. [20], Crosby et al. [21], Ai et al. [22], and Wammack et al. [23] obtained the deposition states of the long-term deposition processes in actual engine working environments using short-term high-concentration particle injection conditions. To create a deposition state of the high-pressure turbine blade surface similar to that under real-engine working conditions on the test bench, the design of the test bench in this method matched the flow conditions of a real engine combustion chamber outlet. Crosby et al. [21] studied the effects of particle diameter, gas temperature, and wall temperature on particle deposition on the TADF experimental bench. It was found that, when the particle diameter was 3–16 μm, the particle capture amount increased with the increase in diameter. The particle capture amount decreased with the decrease in gas temperature. For the influence of wall temperature on deposition, a flat plate with impact cooling on the back was used to simulate the deposition characteristics of the outer surface of the real hollow blade wall with internal cooling. The results showed that, with the increase in cooling air volume, wall temperature decreased and the amount of particle trapping also decreased. Ai et al. [22] also further studied particle deposition of the discrete hole film cooling circular plate on the TADF test bench. The deposition decreased with the increase in blowing ratio. In the downstream area of the film hole outflow, the deposition amount was lower, while between the film holes, the deposition amount was higher. Wammack et al. [23] studied the dynamic variations of deposition morphologies on a polished wall, a wall with a polished coating, and a rough coating wall with time on the TADF test bench. The experimental results showed that the wall deposition morphologies of the three different surface treatments were similar. Casaday et al. [24] numerically simulated the effect of hot spots on the particle deposition of uncooled turbine cascades. The hot spots were simulated as Gaussian distributions of the upstream temperature of the blade, and the critical viscosity model was used to predict particle deposition. The results showed that the higher the surface temperature, the higher the degree of particle deposition on the blade. Prenter et al. [25] studied the effect of an uneven inlet temperature distribution on particle deposition of the guide vane, with split film cooling. The results showed that inlet temperature distribution and film cooling had a significant influence on particle capture efficiency, and the deposition distribution was also affected by the temperature change of the blade surface.

The research on the particle deposition behavior in divergent cooling has mainly focused on the influence of flow field conditions, physical properties of the main-stream and cooling working fluid, and material parameters of the divergent cooling matrix on divergent cooling. The few studies on divergent cooling flow field and particle motion did not consider the deposition of particles on the wall, and there has been no research on the deposition characteristics of particles on the divergent cooling wall. In this study, the interactions between particle deposition and divergent cooling were studied experimentally, and the particle deposition mechanism of the divergent cooling wall was analyzed by numerical simulations. The influence of particle deposition on the divergent cooling was revealed; that is, with the same values of particle parameters, main-stream parameters, and initial blowing ratio, the evolution of deposition characteristics with time was systematically analyzed. With the real sintered metal mesh cooling structure, the particle deposition under a nearly real divergent cooling flow field was studied by numerical simulations.

## 2. Experimental Setup

### 2.1. Experimental System and Test Sections

**Figure 2:**
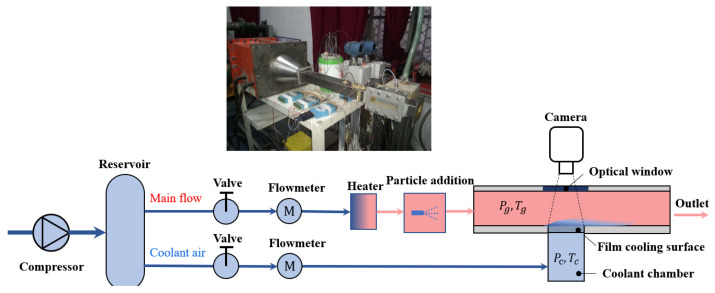
Overall layout of the experimental system.

In this study, a divergent cooling particle deposition experimental platform was built, as shown in Figure 2. The experimental platform mainly included the following systems: a gas supply system (which supplied the mainstream and cold air flow), a heating system, an experimental section system, a temperature and pressure measurement system, and a data acquisition system.

The air compression system adopted a DJ150 A 110 kW screw air compressor. The volume flow rate was 20.5 m^3^/min, and the exhaust pressure was 0.8 MPa. The high-pressure air provided by the compressor flowed through the filter element type gas–liquid separator, coalescing filter oil remover, and other equipment, and was stored in a high-pressure gas storage tank, which would ensure the stable supply of high-pressure gas within a certain period of time. One end of the heater was the inlet part, and the other end was the outlet part. To prevent heat dissipation, the outlet pipe was wrapped with cotton as an insulation material, with a thickness of cotton of 5 mm and a thermal conductivity of 0.03 W/m∙K. The power supply voltage of the heating device was up to 380 V, the maximum heating power was 125 kW, and the maximum heating temperature was 50 °C. In the experimental section system, because the shooting size of the designed experimental section was much smaller than the size of the heater, in order to ensure the smooth progress of the experiment, the connecting part between the heater outlet section and the experimental section was designed as the contraction section. The main rectangular channel of the experimental shooting section was 30 mm wide, 45 mm high, and 495 mm long. The experimental shooting section was located at the tail of the main-stream channel. The size of the infrared window was the same as that of the experimental section, and the dimensions were 90 mm × 30 mm × 6 mm. After the cooling air entered the cooling chamber and was fully mixed, it flowed through the inside of the experimental piece and flowed out from its outer surface. The infrared thermal imager measured the surface temperature of the experimental piece through the infrared window on the other side. Except for the porous wall temperature, all temperatures were measured by a K-type thermocouple, which was calibrated (±0.1 K accuracy) before the experiment. All pressures were monitored using a Rosemont sensor (accuracy of 0.15%). The analog signals of temperature and pressure were converted into digital signals by the ADAM-4018 chip and then recorded on the computer.

**Figure 3:**
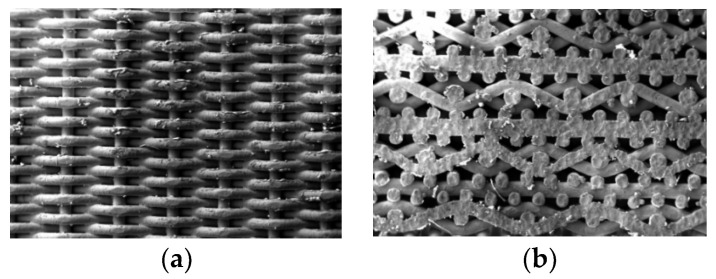
Surface structure of sintered metal wire mesh: (**a**) sintered metal wire mesh outer surface and (**b**) sintered metal wire mesh cross section.

**Figure 4:**
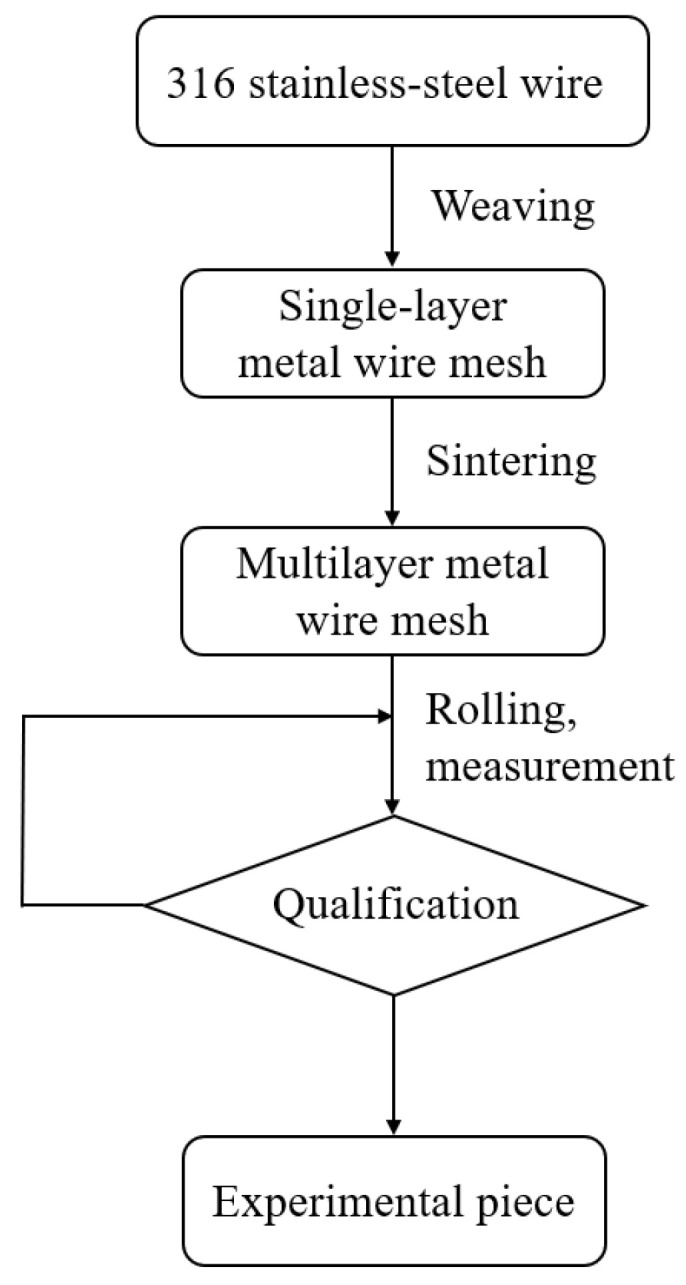
Sintered metal wire mesh processing.

**Figure 5:**
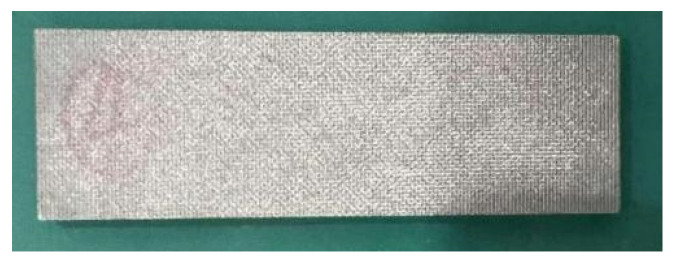
Plate test piece used in the experiment.

The sintered metal wire mesh experimental pieces used in this study were manufactured by Beijing Jingming Powder Metallurgy Co., Ltd. in Beijing, China, and were made of twill dense metal wire mesh. The main reason was that its structure was compact, there were many solder joints, and its strength was better than those of other woven forms of wire mesh. Figure 3 shows the surface structure of the mesh obtained by scanning electron microscopy. The processing of the sintered metal wire mesh is shown in Figure 4. The raw material of the experimental mesh was 316 stainless-steel wire. The metal mesh was woven into a single-layer mesh by Dutch mesh weaving, and then the multi-layer mesh was interlaced and sintered in a vacuum high-temperature furnace to form a multi-layer sintered metal mesh. Through the rolling process, plastic deformation and thickness were reduced, and porosity and average pore size were also reduced. By continuously adjusting the magnitude of the applied load during the rolling process, the sintered metal wire mesh with the required parameters was finally obtained. The sintered metal mesh used in this study had 20 layers, the porosity was 55.1%, and the average pore size was 93.7 μm. Figure 5 shows the plate test piece used in this experiment.

**Figure 6:**
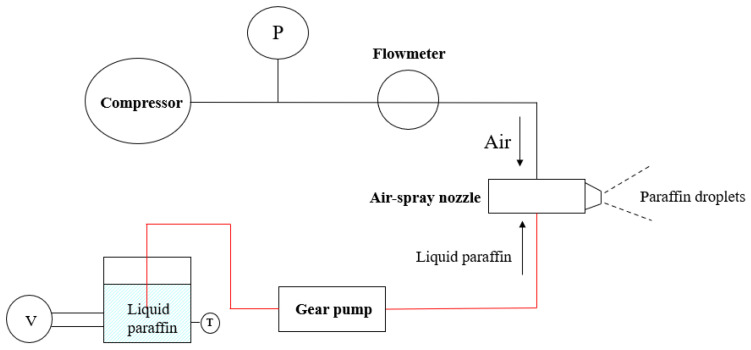
Particle injection system.

The phase-change energy storage material, paraffin, was selected as the target particle for the particle deposition experiments to simulate particles in the flow channel of the aero-engine turbine system, and the corresponding particle injection system was designed, as shown in Figure 6. The particle injection system included a jet device, an air pipe, and a liquid pipe (the red line in the figure). The liquid path was used to transport the liquefied paraffin to the air atomizing nozzle through the gear pump, while the gas path provided the compressed air required to atomize the liquid paraffin. The liquid paraffin and compressed air converged in the air atomizing nozzle and then ejected the paraffin particles in the form of droplets.

**Figure 7:**
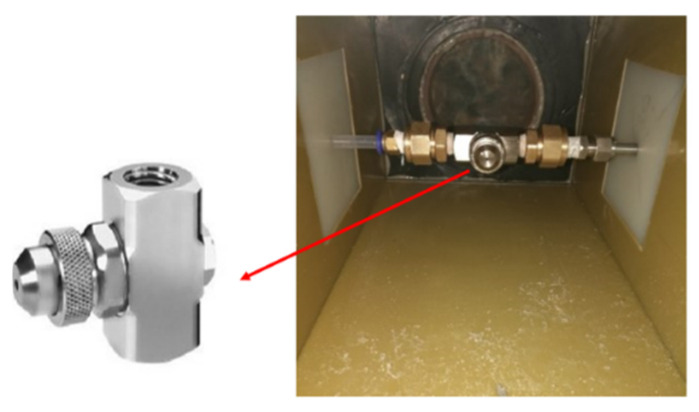
Installation method of the nozzle in the main-stream channel.

In the process of the spray experiment, due to heat loss during the process of paraffin flowing through the pipeline, in order to prevent paraffin solidification, some pipelines were designed as copper pipes, with a heating film on the outer wall of the copper pipe to ensure the patency of the flow channel. Moreover, the gas path of the spray system was pre-mixed with paraffin liquid inside the nozzle. To prevent paraffin from solidifying and blocking the nozzle on the inside, copper pipes were also installed in the gas path pipeline and attached to the heating film. By preheating the air, the circulation of the whole spray system was ensured during the experiment. In this experiment, the air atomizing nozzle produced by Spry Spray System Co., Ltd. in Shanghai, China was selected, as shown in Figure 7. A pressure spray device was formed by pipeline connections. By adjusting the gas–liquid mass flow ratio, the liquid paraffin could be separated into particles with the sizes required for the experiment. Paraffin (solid at room temperature) was stored in a pressure tank and melted by a heating device. The liquid paraffin was pumped into the copper tube from the pressure tank by a gear pump and flowed into the nozzle. It was mixed with the hot air transported by the gas path inside the nozzle and was sprayed into the main stream after being atomized by the nozzle. The installation position of the air atomization nozzle in the main-stream channel is shown in Figure 7. The particle size produced by the spray system was measured by a Malvern particle size meter and was mainly two sizes, 20.75 μm and 55 μm.

**Figure 8:**
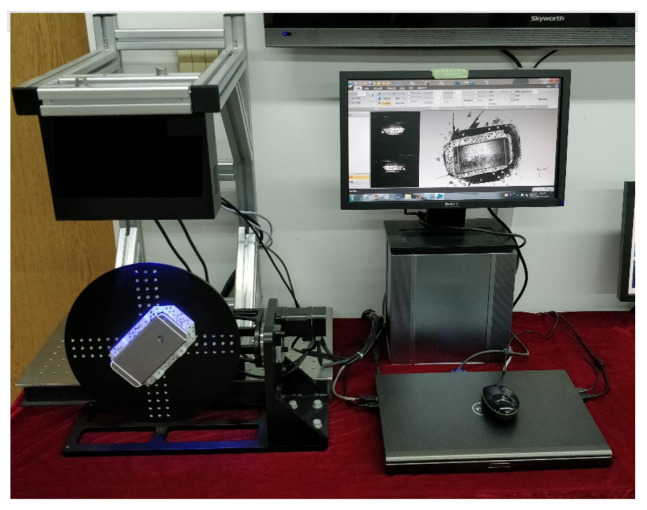
Binocular grating projector.

To obtain the distribution of particle deposition on the divergent cooling wall, a binocular grating projector was used to measure the plate and obtain the spatial distribution of the particle deposition. The principle of the device was mainly based on the stereo-vision method of shape recovery based on the principle of dual-view geometry. The images collected by two cameras from different perspectives were used for feature extraction and stereo-matching. Finally, the binocular parallax principle was used to restore the shape of the object. The main steps of the binocular grating projection were camera calibration, projection grating image acquisition, grating image phase unwrapping, feature point matching, and reconstruction of the three-dimensional point cloud based on the epipolar geometry principle. Figure 8 shows a photograph of the binocular grating projector.

### 2.2. Experimental Procedure

In the experiment, the particle injection system was not opened at the beginning. First, the stable divergent cooling flow field and temperature field were obtained. The flow parameters required for the experiment were obtained by opening and adjusting the gas path valve and observing the flowmeter. By changing the supply voltage and current of the electric heater, the main-stream temperature required for the experiment could be achieved. The pressure and temperature of the main flow and coolant were used to observe whether the flow was stable. When the temperature of the porous wall was stable, the image file was recorded. Before the molten paraffin was sprayed onto the test section, the flow field parameters were kept in a stable state for at least 5 min so that the model could reach a thermally stable state. Then, the particle injection system was turned on to perform particle deposition experiments, and the timing began. By weighing the wax tank before and after a spray process, the net quality of the wax used in each spray event was obtained. The average wax mass flow rate of the spray event was usually about 10 g/min. The deposition quality after the deposition experiment was determined by removing the divergent cooling test piece from the test bench after closing the particle injection system, weighing the test piece, and comparing it with the test piece before deposition to obtain the deposition quality for a specific time period. The deposition thickness distribution on the surface of the entire experimental piece was obtained by scanning the deposited divergent cooling experimental piece through a grating projector.

## 3. Numerical Setup

In this paper, the Fluent discrete phase model (DPM) was used to simulate the particle deposition. This method can calculate the trajectories of particles using Newton’s second law under the Lagrangian framework, by analyzing the force of the fluid on particles. Moreover, it can also take into account the heat/mass transfer caused by particles and the influence of the particle phase and fluid phase coupling on the particle trajectory and fluid flow. The initial conditions of the particle injection were obtained by defining the coordinates, velocities, diameters, and particle temperatures at injection and setting the physical characteristic parameters of the particles. On this basis, the Fluent discrete phase model can be used to simulate the track and heat/mass transfer of the initial particles. The movement of particles in the fluid was based on the force exerted by the local fluid on the particles, and the heat and mass transfer was driven by the convection and radiation between fluid particles. Thus, the heat and mass transfer between the particle track and the particle fluid could be realized.

The motion of a solid particle in a fluid is described by the Lagrangian method, and its position and velocity at each time step can be calculated by means of an ordinary differential equation. This equation is known as the Basset-Boussinesque-Oseen (BBO) equation and is a reflection of Newton’s second law. The general form of the BBO equation is shown below:

(1) $$m_{p}\frac{du_{p}}{dt}=F_{D}+F_{\mathrm{AM}}+F_{B}+F_{\mathrm{PG}}+F_{\mathrm{BA}}+F_{S}+F_{\mathrm{other}}$$

Here is the particle mass andthe particle velocity, and t is the time of particle motion. On the right-hand side of the equation are the forces on the particle moving in the fluid, including fluid drag, additional mass forces, volume forces (gravity and buoyancy), pressure gradient forces, Basset Force, Saffman force, and various other forces.

Whereas for current particle deposition studies, the near-wall flow field is the focus of attention, the large velocity gradient within the velocity boundary layer requires consideration of the effect of the Saffman force on particle motion and, therefore, Equation (1) can be simplified as:

(2) $$m_{p}\frac{du_{p}}{dt}=F_{D}+F_{S}=-\frac{1}{8}\pi d_{p}^{2}\rho_{f}C_{D}(u_{f}-u_{p})\left|u_{f}-u_{p}\right|+\frac{3.1(u_{f}-u_{p})\sqrt{\rho_{f}\mu \left|du_{f}/dy\right|}}{\rho_{p}d_{p}}$$

where $u_{p}$ and $u_{f}$ are the particle and gas phase velocities, $\rho_{p}$ and $d_{p}$ are the particle density and diameter, respectively, $C_{D}$ and is the drag coefficient.

For a complete calculation of the particle trajectory, it is also necessary to include the particle displacement equation and the particle angular velocity equation, and the complete particle motion is described by the following equations:

(3) $$\left\{\begin{array}{l} \frac{dx_{p}}{dt}=u_{p}\\ m_{p}\frac{du_{p}}{dt}=F_{D}+F_{S}\\ I_{p}\frac{d\omega_{p}}{dt}=T \end{array}\right.$$

where $x_{p}$ is the particle position, $I_{p}$ is the particle moment of inertia, $\omega_{p}$ is the particle angular velocity, and T is the torque applied to the rotating particle in the fluid. When the rotation of the particle itself is not considered, the equation of motion of the particle is simplified as:

(4) $$\left\{\begin{array}{l} \frac{dx_{p}}{dt}=u_{p}\\ m_{p}\frac{du_{p}}{dt}=F_{D}+F_{S} \end{array}\right.$$

Applications of two-phase flow usually involve turbulence, which must be taken into account in the calculation of particle trajectories based on its direct effect on particle motion. Due to the natural fluctuating nature of the turbulent velocity field, a dispersion effect is observed for particles released from the same location, i.e., there are different trajectories for particles released from the same location at different times. This phenomenon is called turbulent diffusion. When considering the effect of turbulent diffusion on particle motion, the instantaneous velocity of the turbulence needs to be known when particle trajectories are calculated using particle motion equations. However, information about these instantaneous fields is not provided when solving turbulent fields using the RANS method. Therefore, depending on the turbulence model used, turbulent diffusion also needs to be modeled. In this study, the Discrete random walk model (DRW) method was chosen to model the turbulent diffusion characteristics of particles.

**Figure 9:**
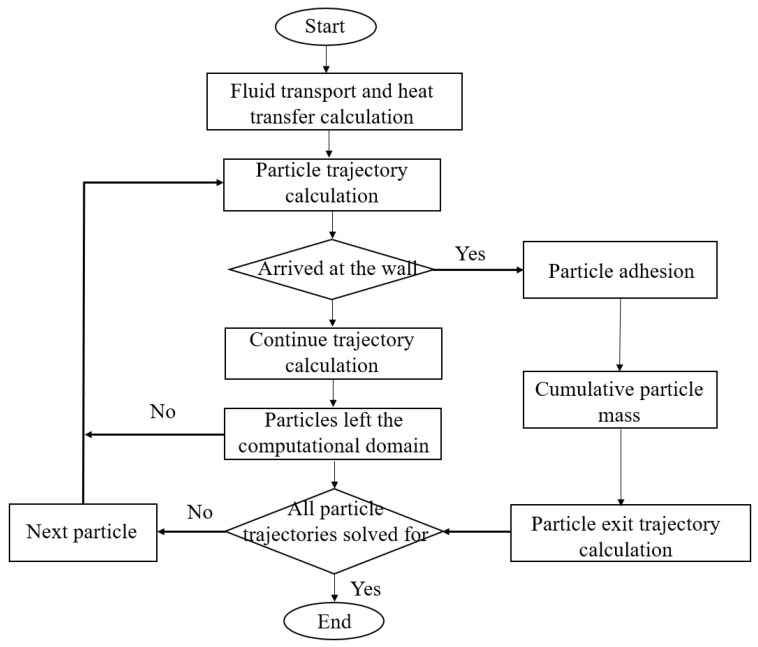
Particle deposition calculation process.

Based on the calculation process shown in Figure 9, the particle deposition simulation was completed. The specific process is as follows:

(1) Based on the geometric model, the flow and heat transfer model was solved to expand the flow field calculation.
(2) After the convergence of the flow field calculation, the particle trajectory was calculated. When the particle moved to the wall surface, it was considered to be captured by the wall surface, and the particle mass was accumulated. If the particle did not reach the wall surface, the particle trajectory was calculated.

If a particle precipitated on the wall or left the computational domain, the trajectory calculation of the particle was stopped and that of the next particle was performed until all trajectory calculations of all particles were complete.

## 4. Results and Discussion

### 4.1. Time Evolution of Particle Deposition Characteristics

When the total particle injection mass was constant, the deposited masses on the divergent cooling wall for two particle mass flow rates were compared. The experimental parameters and deposited masses are shown in Table 1.

**Figure 10:**
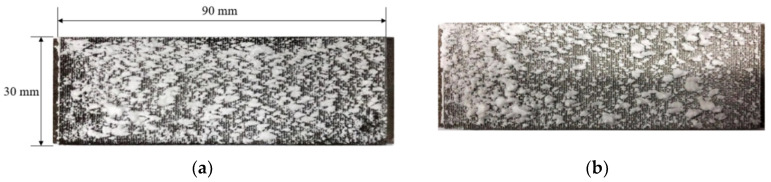
Deposition distribution at different particle concentrations: (**a**) Experiment 1 and (**b**) Experiment 2.

Figure 10 shows the deposition distributions for two particle concentrations when the total particle injection amount was constant. It can be seen that although the total particle injection amount was the same on the current divergent cooling wall, deposition distributions were quite different. At the large particle concentration (Experiment 1), more large deposition blocks formed on the wall, and the distribution of deposition blocks was dense, as shown in Figure 10a. At the low particle concentration (Experiment 2), as shown in Figure 10b, the distribution of sedimentary blocks was relatively sparse. Combined with the deposition results of Table 1 and Figure 10, it is also shown that the deposition amount with a high particle concentration was larger on the current divergent cooling wall.

It can be seen from the results that the equivalent acceleration effect of the particle deposition was not always satisfied, and the influence of particle concentration and deposition time needed to be considered at the same time.

The blowing ratio F, which measures the mass flow ratio between the cooling air and hot air in the main stream [26], is defined as follows:

(5) $$F=\frac{\rho_{c}u_{c}}{\rho_{g}u_{g}},$$

where *ρ_c_* and *ρ_g_* are the densities of the coolant and main flow, respectively, and *u_c_* and *u_g_* are the velocities of the coolant in the cooling chamber and main flow, respectively.

**Figure 11:**
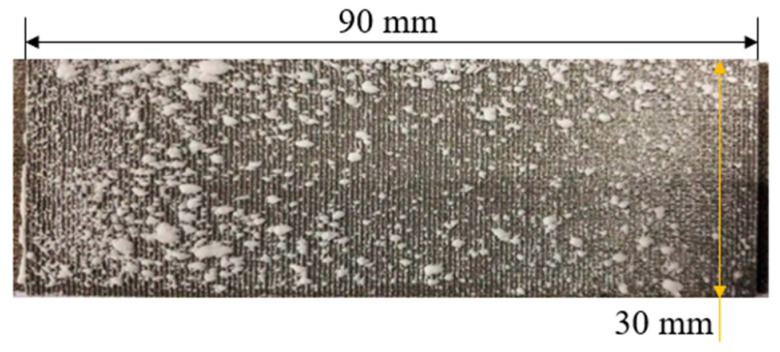
Experiment A: spray time of 2 min.

**Figure 12:**
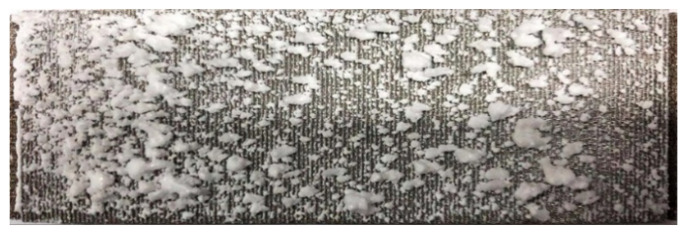
Experiment B: spray time of 3 min.

**Figure 13:**
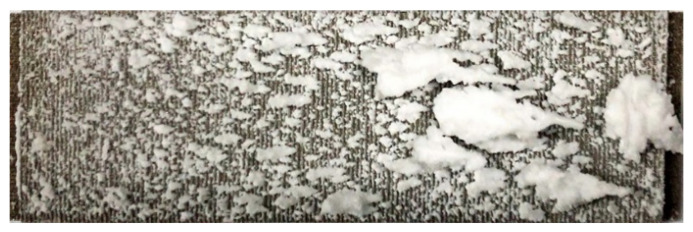
Experiment C: spray time of 5 min.

The evolution of the deposition state with the increase in the spray time was evaluated by comparing Experiments A, B, and C. The experimental conditions are shown in Table 2. Figure 11, Figure 12 and Figure 13 show the deposition distributions of the sintered metal wire mesh divergent cooling wall for different spray times. It can be seen that deposition distributions were similar, while deposition thicknesses were significantly different. When the spray time was short, as shown in Figure 11, the deposition was relatively thin, and the deposition amount upstream of the divergent cooling wall was greater than that downstream. As the spray time increased, the deposition thickness of the entire wall increased significantly. As shown in Figure 12, the deposition thickness in the downstream area was closer to that in the upstream area. When the spray time reached 5 min, as shown in Figure 13, many thicker sedimentary blocks were formed, and the coverage area of the sedimentary blocks was significantly increased. For the results of Experiment C, Figure 13 shows that there was a large deposition accumulation downstream of the divergent cooling wall, but not upstream. In fact, under the conditions of a 5 min spray, there were also a large number of large deposition blocks on the upstream divergent cooling wall. However, due to the thickness of the deposited layer being too large, it was peeled off when the experimental part was disassembled, leading to the results shown in Figure 13.

**Figure 14:**
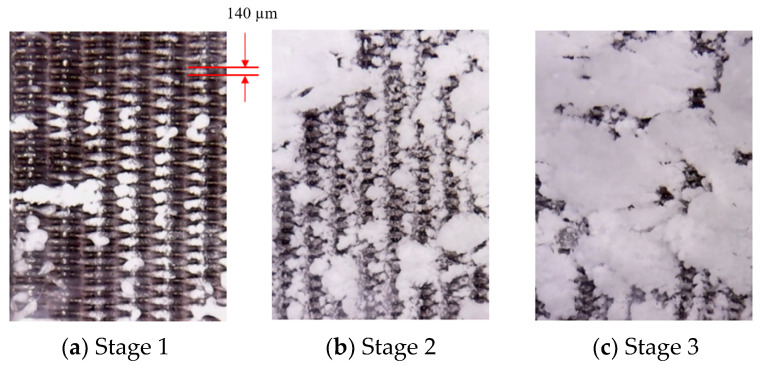
Deposition state evolution with time, amplified by 20×.

**Figure 15:**
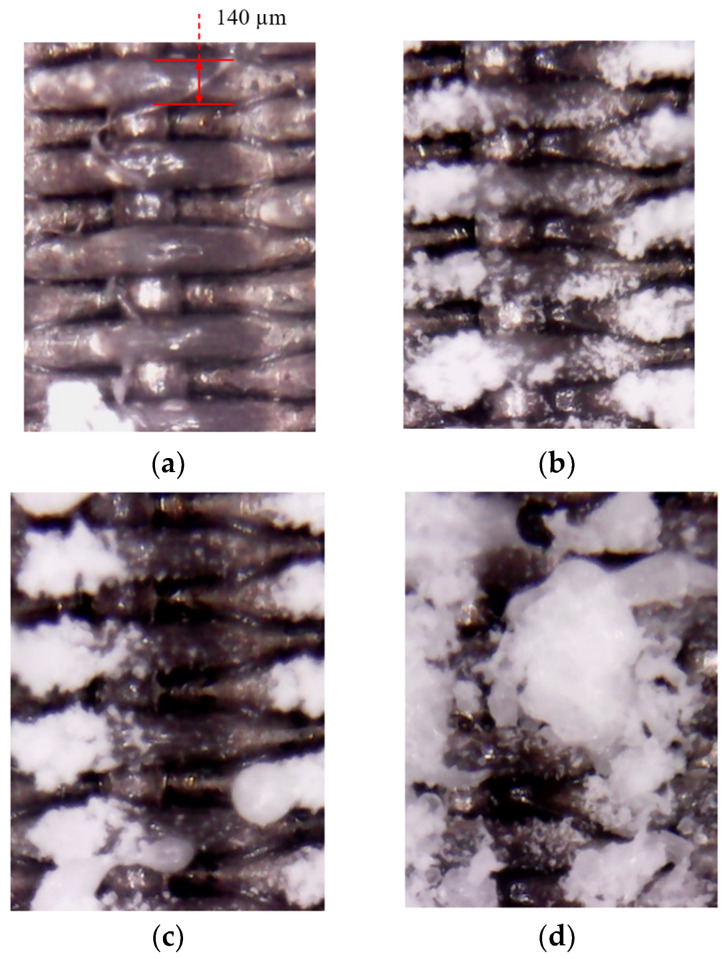
Pore-scale deposition morphology evolution over time, amplified by 30×. (**a**) No deposition, (**b**) main-stream windward deposition, (**c**) depositional extension in spanwise direction, and (**d**) depositional spanwise connection.

The formation of large deposition blocks on the divergent cooling wall can be observed from the pore scale. Figure 14 and Figure 15 show the evolution of the deposition state of the sintered metal wire mesh wall, with time under different magnifications. It can be seen that, as spray time increased, large deposition blocks were formed by the gradual increase in the amount of sediment on the adjacent metal wires and the fusion after contact. In the initial stage of deposition, particles could easily deposit on the upper surface of the windward side of the metal wire mesh, as shown in Figure 15b. As spray time increased, the deposition on the surface of the metal wire increased, and the deposition on the windward side of the metal wire gradually diffused from the center to both sides, as shown in Figure 15c. When the spray time continued to increase, the deposition blocks on the surface of the metal wire extended to both sides, connected with the deposition blocks of adjacent metal wires, and gradually merged together to form a large area of deposition blocks, as shown in Figure 15d.

**Figure 16:**
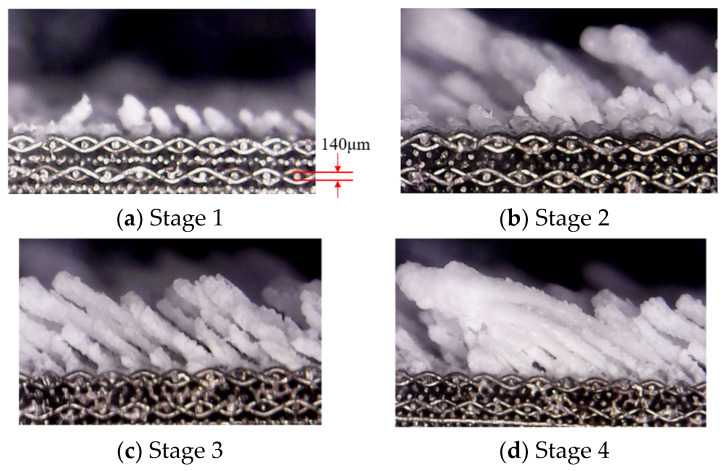
Time evolution of sedimentary growth from a horizontal perspective.

Figure 16 shows the evolution of deposition growth over time from the perspective of the cross section of the divergent cooling wall. The main-stream flow direction was from left to right in the figure. It can be seen that the growth direction of the sediment on the surface of the sintered metal mesh followed certain rules, and almost all of them grew in the opposite direction of the main stream. Combined with the results in Figure 15, the particles were most likely to deposit on the windward side of the metal wire. When the particles increasingly accumulated in this area, deposition thickness gradually increased. Due to the geometric relationship between the windward side of the metal wire and the main stream, sediment accumulated and grew along the windward side in an approximately vertical direction, forming in the reverse direction of the main stream from a horizontal perspective.

It can also be seen from Figure 16 that the formation of large deposits on the wall of the sintered metal wire mesh was not only due to the gradual increase in the deposits on adjacent metal wires perpendicular to the flow direction but was also related to the gradual fusion of deposits on two adjacent winding units of metal wires in the flow direction. The counter-main-stream growth of downstream sediment was increasingly inclined downward due to the motion of the sediment, so that it was increasingly closer to the upstream sediment. As spray time increased, upstream and downstream sediments eventually merged together.

### 4.2. Variation of Particle Deposition Quality and Thickness with Time

**Figure 17:**
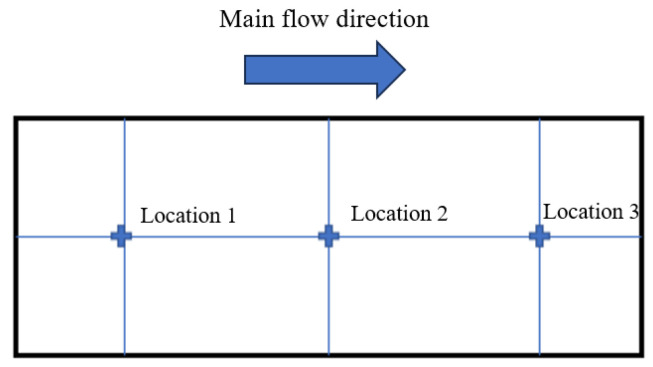
Deposition thickness measurement positions of the divergent cooling wall.

**Figure 18:**
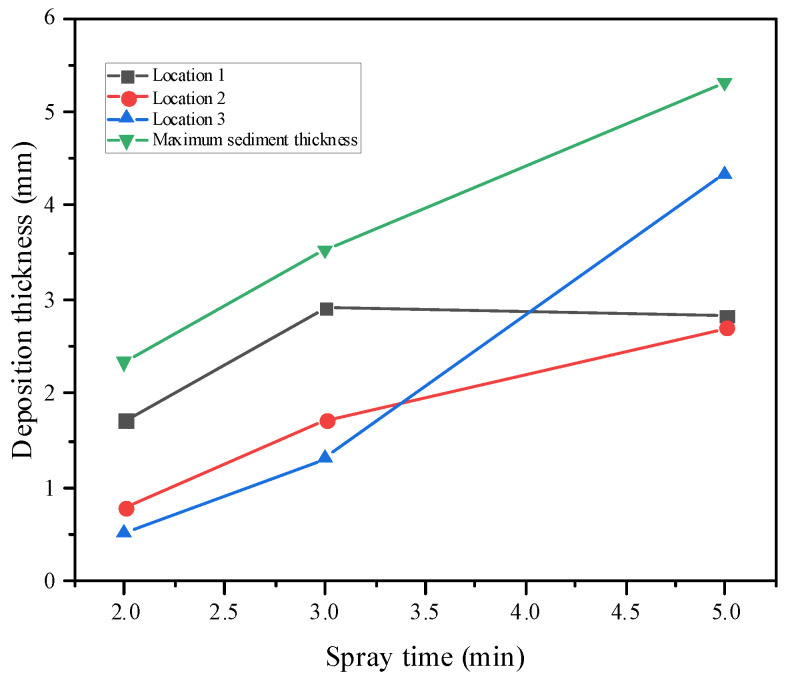
Variation of deposition thickness with time at each position.

For the three sets of experiments, 5 mm × 5 mm areas were selected to measure the deposition thicknesses at three positions on the divergent cooling wall, as shown in Figure 17. These deposition thickness measurements are plotted in Figure 18. Moreover, the maximum deposition thickness of the wall after different spray times was also measured. The results showed that sediment thickness increased with an increase in the spray time, which was approximately linear. Position 1 was upstream of the divergent cooling wall, and the deposition thickness was the greatest in this area. The deposition thickness at position 3 in the downstream area was much lower than that in the upstream area when spray time was short, but as spray time became increasingly longer, the deposition thickness at positions 1 and 3 became closer. It can also be seen from the statistics of the maximum deposition thickness that the thickness of the sediment increased with the increase in spray time. The deposition thickness at position 1 decreased after a long time because of shedding of the deposition block.

**Figure 19:**
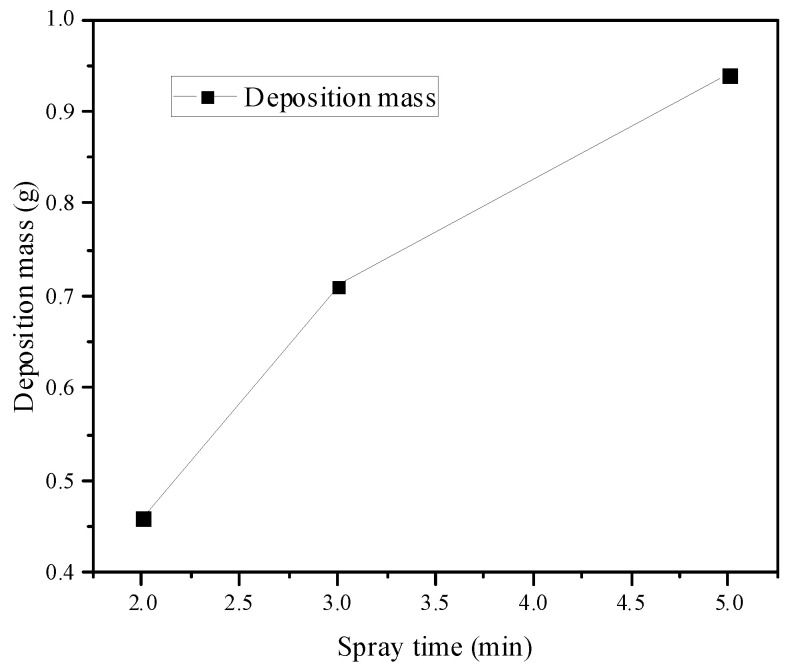
Variation of deposition mass with time.

**Figure 20:**
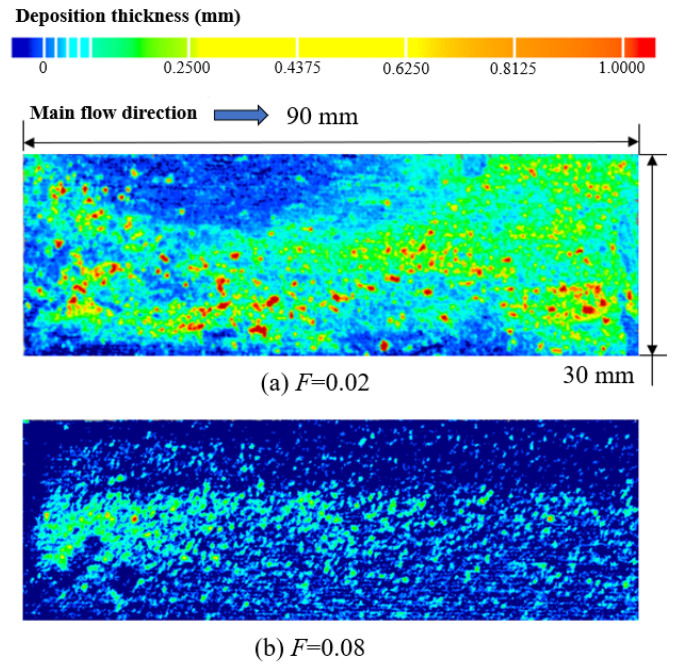
Particle deposition distribution on the surface of the test piece after a spray time of 1 min.

**Figure 21:**
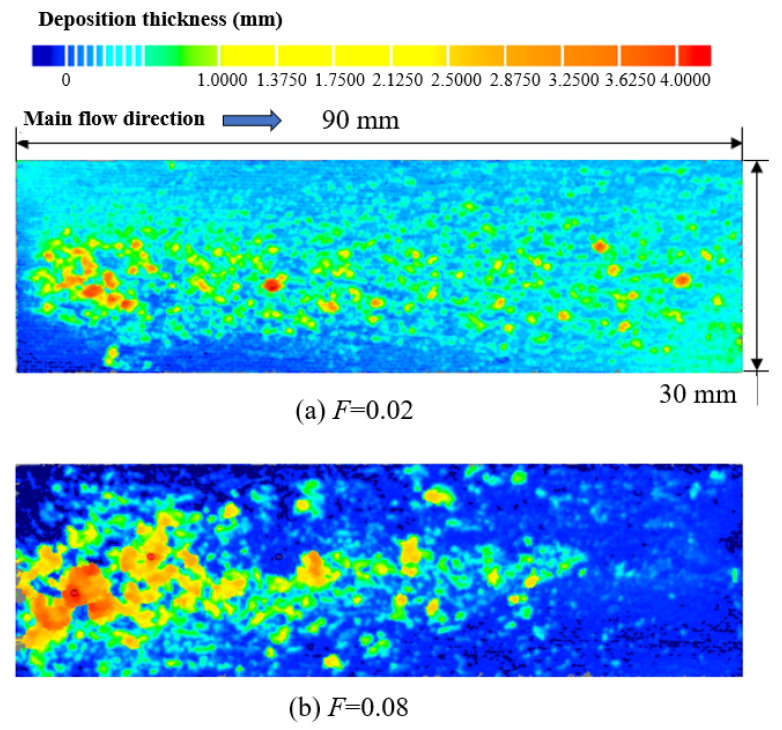
Particle deposition distribution on the surface of the test piece after a spray time of 3 min.

The variation trend of deposition mass with spray time is shown in Figure 19. It can be seen that, under the current conditions, deposition mass continued to increase with the spray time. The distribution of particle deposition on the divergent cooling wall under typical conditions is shown in Figure 20 and Figure 21. It can be seen from these images that there was a certain regularity in the distribution of particles on the surface of the metal mesh. When there was cooling air flow, there were more deposits on the leading edge of the metal mesh, which tended to decrease gradually in the direction of the main stream. At the same time, the sediment gradually decreased along the central axis of the wire mesh to both sides, and the overall distribution was a “∩” type, which is also similar to the spanwise temperature distribution of the divergent cooling wall without deposition.

**Figure 22:**
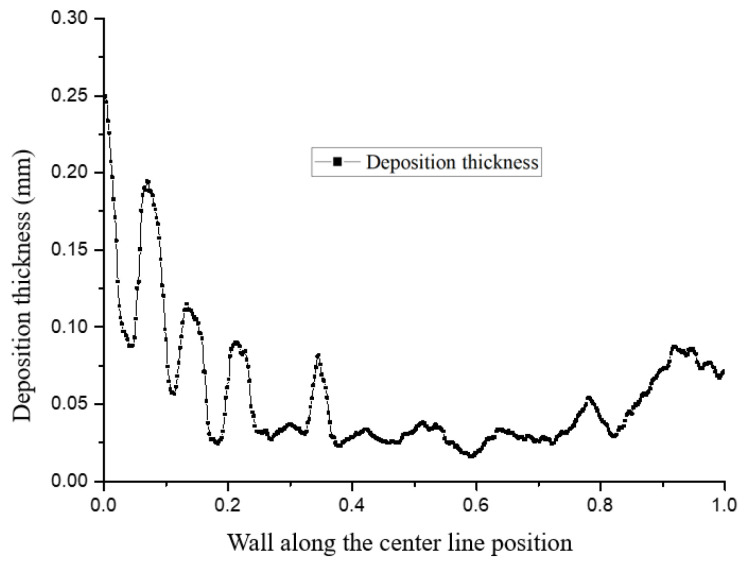
Particle deposition thickness on the surface of the test piece along the neutral line after a spray time of 1 min.

**Figure 23:**
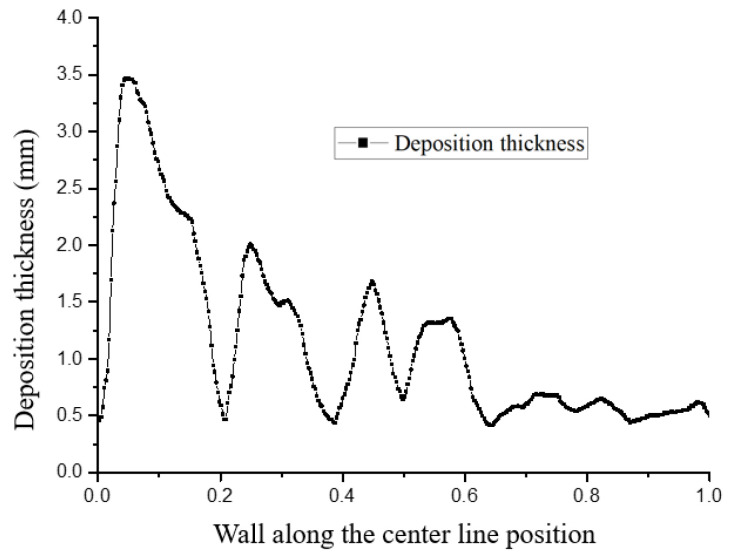
Particle deposition thickness on the surface of the test piece along the neutral line after a spray time of 3 min.

Figure 22 and Figure 23 show the distribution trends of the deposition thickness along the center line of the divergent cooling wall after two spray times. It can be seen that, with the increase in spray time, the distribution of the particle deposition was closer to the temperature distribution on the surface of the metal mesh. This was because the cooling air flow on the surface of the mesh covered the surface of the metal mesh to form a gas film under the action of the main stream. The leading edge of the metal mesh was the first to contact the main stream, and the cooling air flow was the smallest at the leading edge position. Along the main stream direction, the cooling air flow gradually accumulated, the gas film gradually thickened, and the cold protection effect of the mesh was more evident. The molten particles in the main stream were more viscous at higher temperatures and were more likely to adhere to the surface of the metal mesh and deposit. Further, due to the thicker gas film at the back of the screen, the resistance to particle deposition was greater. Under the combined influence, the deposition thickness was smaller than that of the front edge of the screen.

It can also be seen from Figure 22 and Figure 23 that there were significant fluctuations in the deposition thickness distribution of the divergent cooling wall. The divergent cooling test piece in this study was a sintered metal woven mesh, which was a porous metal material with complex and interconnected pore channels. After deposition, the deposition layer covered the cooling hole of the metal braided mesh. The deposition coverage of the divergent cooling wall was not formed at the same time but gradually became uniform with the deposition process, transitioning from dispersed to uniform. The difference in deposition thickness caused by the uneven distribution of deposition in adjacent areas led to a significant difference in the degree of blockage between the two adjacent areas. In areas with thicker sediments, flow resistance was greatly increased. However, in the adjacent downstream area, deposition thickness decreased sharply, so the flow resistance in this area was much smaller. The significant difference in flow resistance led to the redistribution of the cooling medium flow. The cooling medium flow rate through the sedimentary depression area was much higher than that in the upstream severe blockage area and even higher than that in the non-deposition area. The large increase in the cooling air flow rate in this area, in turn, inhibited the deposition of subsequent particles, while in the adjacent upstream area, particles were more likely to deposit, thus causing significant fluctuations in the deposition thickness of the adjacent area.

**Figure 24:**
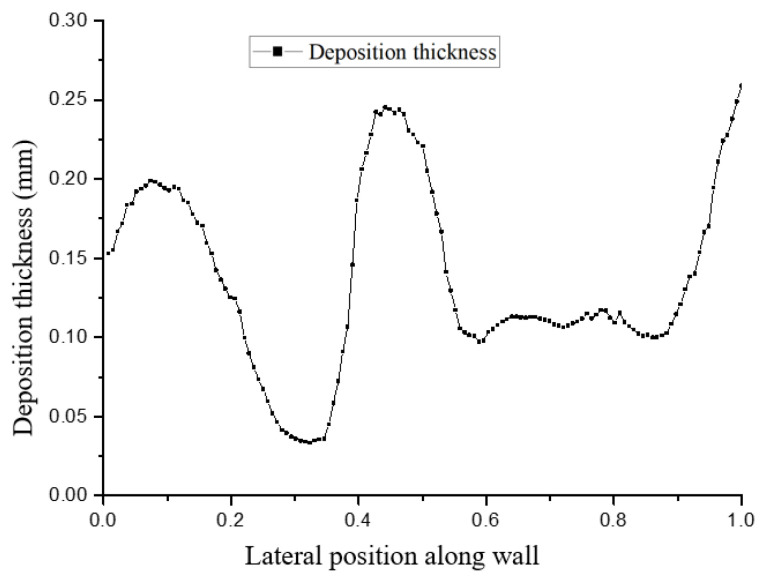
Particle deposition thickness on the surface of the test piece along the spanwise direction after a spray time of 1 min.

**Figure 25:**
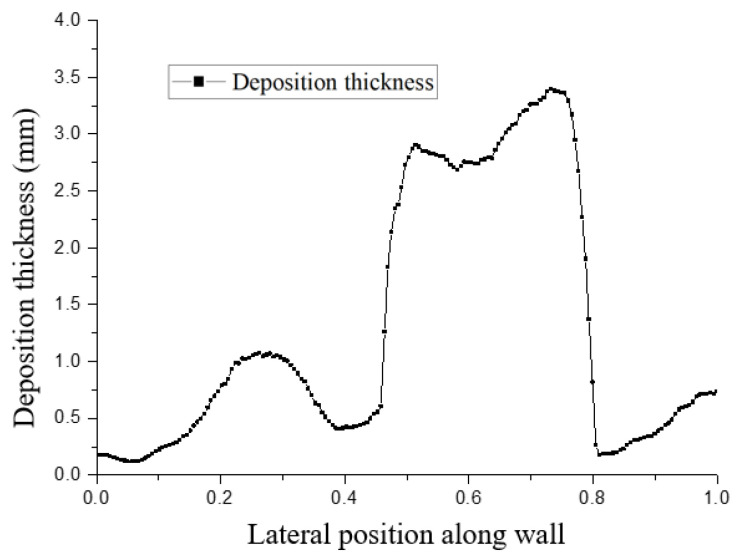
Particle deposition thickness on the surface of the test piece along the spanwise direction after a spray time of 3 min.

Figure 24 and Figure 25 show the distribution of deposition thickness in the spanwise direction of the divergent cooling wall. It can be seen that when spray time was very short, deposition thickness in the spanwise direction was more consistent, and after spray time increased further, the deposition thickness distribution began to form a ∩-type distribution, similar to the temperature distribution of the metal wire mesh wall.

### 4.3. Numerical Simulation of Sintered Metal Mesh Particle Deposition Characteristics Analysis

**Figure 26:**
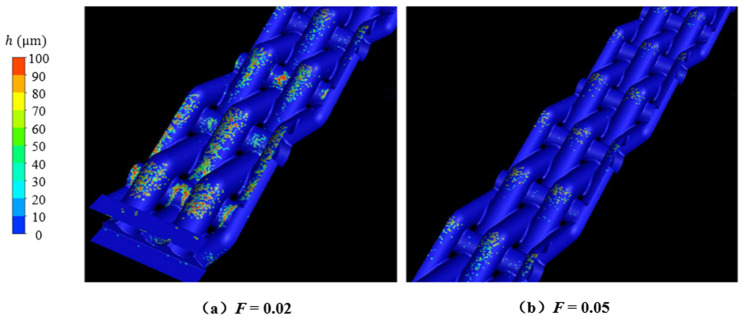
Deposition mass distribution on the surface of metal wire mesh.

**Figure 27:**
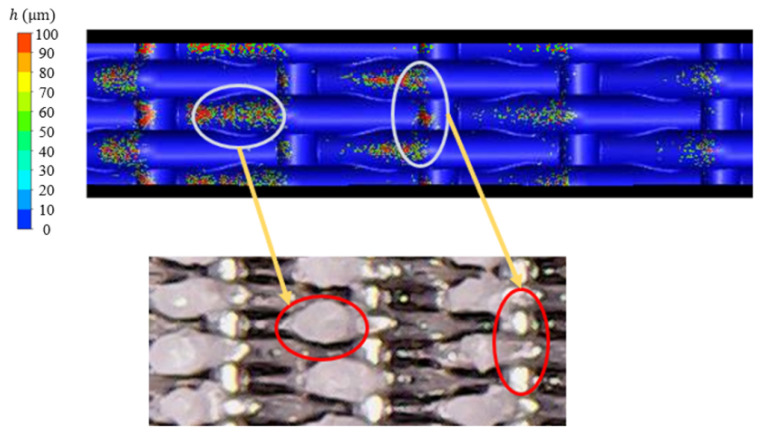
Simulation and experimental comparison of metal wire mesh deposition distribution (The yellow circles indicate the locations where numerically calculated particles were deposited, and the red circle circles indicate the locations where experimentally collected particles were deposited).

Figure 26 shows the deposition mass distribution of the metal mesh wall under two blowing ratios. Figure 27 shows the comparison between the simulation results of the metal mesh particle deposition and the experimental results of the metal mesh deposition. It can be seen that the particles were easily deposited on the windward side of the metal wire in the main-stream direction, which was also consistent with the distribution characteristics of the metal wire mesh deposition obtained in the experiment. The increase in blowing ratio reduced the deposition of particles on the wall of the metal mesh.

**Figure 28:**
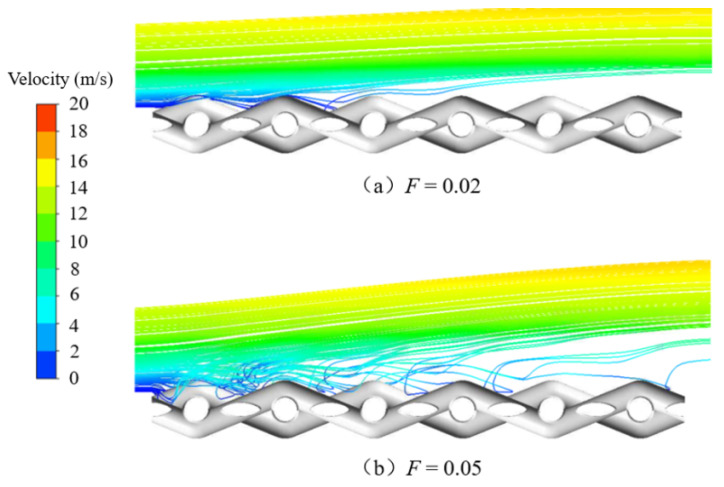
Streamline distribution of the upstream area of the main stream along the wire mesh under different blowing ratios.

**Figure 29:**
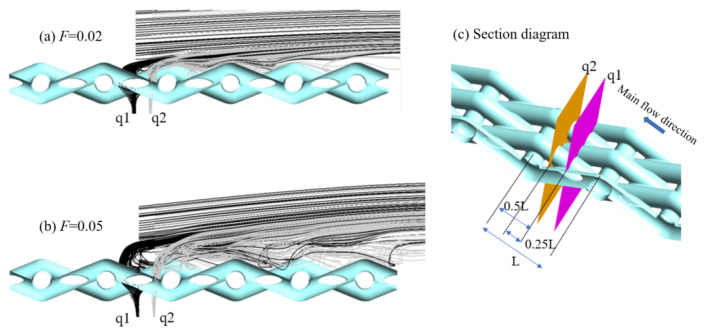
Three-dimensional streamlines of transverse sections p1 and p2 in the middle areas of the metal mesh under different blowing ratios.

When the blowing ratio was small, the cooling film was more adherent, and upstream of the metal wire mesh was the thinnest area of the cooling film. At this time, the main stream could easily directly scour the windward side of the leading edge of the metal wire, as shown in Figure 28. Therefore, the windward side of the most upstream area of the metal wire mesh was the area with the highest deposition. Along the main-stream direction to the downstream region of the metal wire mesh, as the film thickness gradually increased, a more evident cold gas coverage layer formed, and the main stream could not easily act on the metal wire mesh wall directly. Therefore, the amount of particle deposition in the downstream area of the metal wire mesh was lower than that in the upstream area. The particle deposition in the downstream area of the metal mesh was mainly due to interactions of cold gas jets in different pores, as shown in Figure 29. When the blowing ratio increased, the amount of cold air in the upstream area was greater, and the main stream at the leading edge of the metal mesh had relatively greater difficulty directly scouring the wall of the metal mesh. At this time, the mixing of cold air and the main stream was more intense. Therefore, when the blowing ratio was large, the deposition of particles at the leading edge of the metal mesh was affected by both the main stream and the cold air.

**Figure 30:**
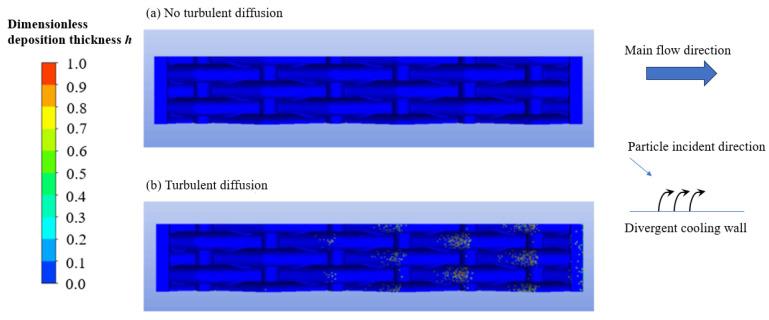
Effect of turbulent diffusion on metal wire mesh deposition.

Figure 30 shows the influence of turbulent diffusion on the deposition of 5 μm particles. The incident direction of the particles was at a certain angle to the divergent cooling wall. Figure 30 shows the deposition distribution without considering the turbulent diffusion; that is, ignoring the influence of turbulent velocity fluctuations on particle motion. At this time, there was no deposition on the wall, indicating that the inertia of the particles themselves was not sufficient to overcome the time-averaged motion of the cold air jet and reach the wall. When the influence of turbulent diffusion was considered, particle deposition could easily occur on the main-stream windward surface of the wire mesh.

## 5. Conclusions

In this study, the divergent cooling technology for aero-engine turbine blades was selected as the research background, and the problem of particle deposition in turbine blades, which needs to be solved in the application of divergent cooling for turbine blade cooling, was explored. An experimental method for simulating the deposition on the divergent cooling wall was designed. Melted paraffin droplets were sprayed into the main stream through an air-atomizing nozzle. These wax droplets deposited and solidified on the divergent cooling wall to simulate the movement of pollutant particles under engine conditions. The research work in this paper can deepen the understanding of interactions between divergent cooling and particle deposition, and provide some theoretical guidance and basic experimental data for the application of divergent cooling technology in the turbine blade local high-temperature zone in the future. Through research, the following conclusions were obtained:

(1) With the increase in spray time, the deposition mass and the maximum deposition thickness of the divergent cooling surface increased gradually.
(2) The distribution characteristics of the deposition thickness on the surface of the metal wire mesh were examined. Along the main-stream direction, when spray time was short, the deposition thickness was higher in the narrow range upstream of the experimental specimen. As spray time increased, deposition thickness gradually decreased along the main-stream direction of the divergent cooling system. In the spanwise direction, when spray time was very short, the deposition thickness in the spanwise direction was more consistent, and when spray time increased further, the deposition thickness distribution began to tend to a ∩-type distribution.
(3) For the metal wire mesh divergent cooling structure, particle deposition could easily occur on the windward side of the metal wire. When the blowing ratio was large, the deposition in the upstream area of the metal wire mesh was mainly due to the mixing of the main stream and the cold air jet, while the deposition of particles in the downstream area was still mainly affected by the cold air jet due to the thick cold air layer.

## Figures and Tables

**Table 1:** Deposition equivalent acceleration experiment of divergent cooling.

Test Times	Spray Time (min)	Particle Mass Flow (g/min)	Deposition Quality (g)
Experiment 1	4	10	2.85
Experiment 2	8	5	1.89

**Table 2:** Experimental conditions for time evolution of sedimentary state.

Test Times	$T_{\mathrm{wax},\mathrm{solid}}$ (K)	$T_{g}$ (K)	ΔT (K)	$T_{c}$ (K)	F	$u_{g}$ (m/s)	$\tau_{\mathrm{spary}}$ (min)
Experiment A	317	315	2	293	0.04	15	2
Experiment B	317	315	2	293	0.04	15	3
Experiment C	317	315	2	293	0.04	15	5

## Data Availability

All processed data in this study are included in this published article. Raw data will be provided on request from the corresponding author.
